# Advancements in Postpartum Rehabilitation: A Systematic Review

**DOI:** 10.7759/cureus.66165

**Published:** 2024-08-05

**Authors:** Asma Shaik, Shahriq Khan, Asra Shaik, Kathijathul Kubra Shaik

**Affiliations:** 1 Internal Medicine, PES Institute of Medical Sciences and Research, Kuppam, IND; 2 General Medicine, Ayaan Institute of Medical Sciences, Hyderabad, IND; 3 Internal Medicine, Government Medical College, Anantapur, Anantapur, IND; 4 Obstetrics and Gynaecology, PES Institute of Medical Sciences and Research, Kuppam, IND

**Keywords:** technology integration, pregnancy, exercises, rehabilitation, postpartum

## Abstract

Postpartum rehabilitation plays a crucial role in promoting maternal health and well-being following childbirth. This systematic review aims to explore recent trends and advancements in postpartum rehabilitation interventions across various categories. A comprehensive search was conducted on PubMed and Embase databases, yielding a total of 358 search results. After applying strict inclusion and exclusion criteria, 20 relevant studies were selected for detailed analysis. These studies were categorized into four distinct categories: exercise-based techniques, technology integration, medical interventions, and multi-modal approaches. Within the exercise-based technique category, pelvic floor exercises, trunk stabilization exercises, and physiotherapy emerged as the most commonly utilized interventions. Studies in this category typically involved population sizes ranging from n = 20 to 200 participants, with study durations spanning from six weeks to 12 weeks. In the technology integration category, predominant techniques included electrical stimulation, biofeedback, acupuncture, and vibrating vaginal balls. Population sizes ranged from n = 50 to 200 participants, with study durations ranging from three days to three months. Only one study was identified in the medical intervention category, which investigated the use of intrathecal analgesia and continuous ropivacaine after cesarean section, involving 200 participants. Multimodal approaches encompassed various combinations of technology, artificial intelligence, image processing, and exercise-based interventions, with population sizes ranging from n = 10 to 500 participants and study durations ranging from six weeks to 12 months. This systematic review provides insights into the diverse landscape of postpartum rehabilitation interventions, highlighting the prominence of exercise-based techniques and the growing utilization of technology integration. While medical interventions remain limited, multimodal approaches show promise in optimizing postpartum care outcomes. Continued research and innovation in this field are warranted to further refine rehabilitation strategies and improve maternal health outcomes following childbirth.

## Introduction and background

The postpartum period, often celebrated for the arrival of new life, stands as a transformative phase in a woman’s journey, marked by intricate physiological changes, diverse challenges, and a profound need for comprehensive rehabilitation [1]. Postpartum rehabilitation, a critical aspect of maternal care, aims to address the unique physical and psychological challenges that women face after childbirth [2,3]. The postpartum period is the fourth trimester encompassing the first few months following delivery and is marked by significant physiological and hormonal changes [4]. During this time, women experience various postpartum conditions such as pelvic floor dysfunction, diastasis recti, musculoskeletal pain, and emotional well-being concerns [5,6]. The severity of postpartum dysfunctions, which greatly affect the quality of life for new mothers, is frequently underestimated and overlooked, despite the significant physical, emotional, and psychological challenges they entail. Recognizing the importance of optimal postpartum recovery, healthcare professionals and researchers have increasingly focused on developing effective rehabilitation strategies tailored to the specific needs of postpartum women.

Postpartum rehabilitation offers a multitude of benefits for women in the post-delivery period, such as musculoskeletal health, emotional well-being, cardiovascular health, weight management, improved sleep, and prevention of long-term complications [2]. The choice of rehabilitation technique depends on various factors. For example, the mode of fetal delivery significantly influences the selection of appropriate rehabilitation techniques tailored to meet the unique needs of postpartum mothers [7]. In cases of normal delivery, interventions often center around pelvic floor exercises and progressive physical activity, aiming to restore musculoskeletal strength and address postpartum physiological changes. Conversely, for cesarean section (C-section) deliveries, a more cautious and phased approach is recommended, emphasizing gentle abdominal exercises and scar tissue management to ensure optimal recovery while minimizing complications [7]. The overall physical health and fitness level of the postpartum woman is also a factor in determining the intensity and progression of rehabilitation exercises. Additional factors, such as medical conditions, lifestyle, nutritional status, social support, and cultural and socioeconomic factors, influence the choice of rehabilitation.

Nowadays, the most common rehabilitation techniques include diastasis recti exercises [3], pelvic floor therapy [8], postural restoration, high-intensity training, and nutritional counseling. In recent years, there has been a substantial increase in research endeavors, including clinical trials and randomized controlled trials, aimed at comprehensively investigating the impacts and outcomes of postpartum rehabilitation [9,10,11]. In this paper, the current landscape of postpartum rehabilitation is explored by examining a diverse range of studies. This review aims to provide insights into the effectiveness of various rehabilitation approaches in addressing postpartum complications and promoting overall maternal well-being. Through a systematic and rigorous analysis of the existing literature, this review aims to contribute to the evidence base, identify gaps in knowledge, and offer recommendations for future research and clinical practice. By shedding light on the current state of postpartum rehabilitation research, this systematic review strives to facilitate informed decision-making, ultimately improving the postpartum experience and long-term health outcomes for women worldwide.

## Review

Methodology

**Figure 1:**
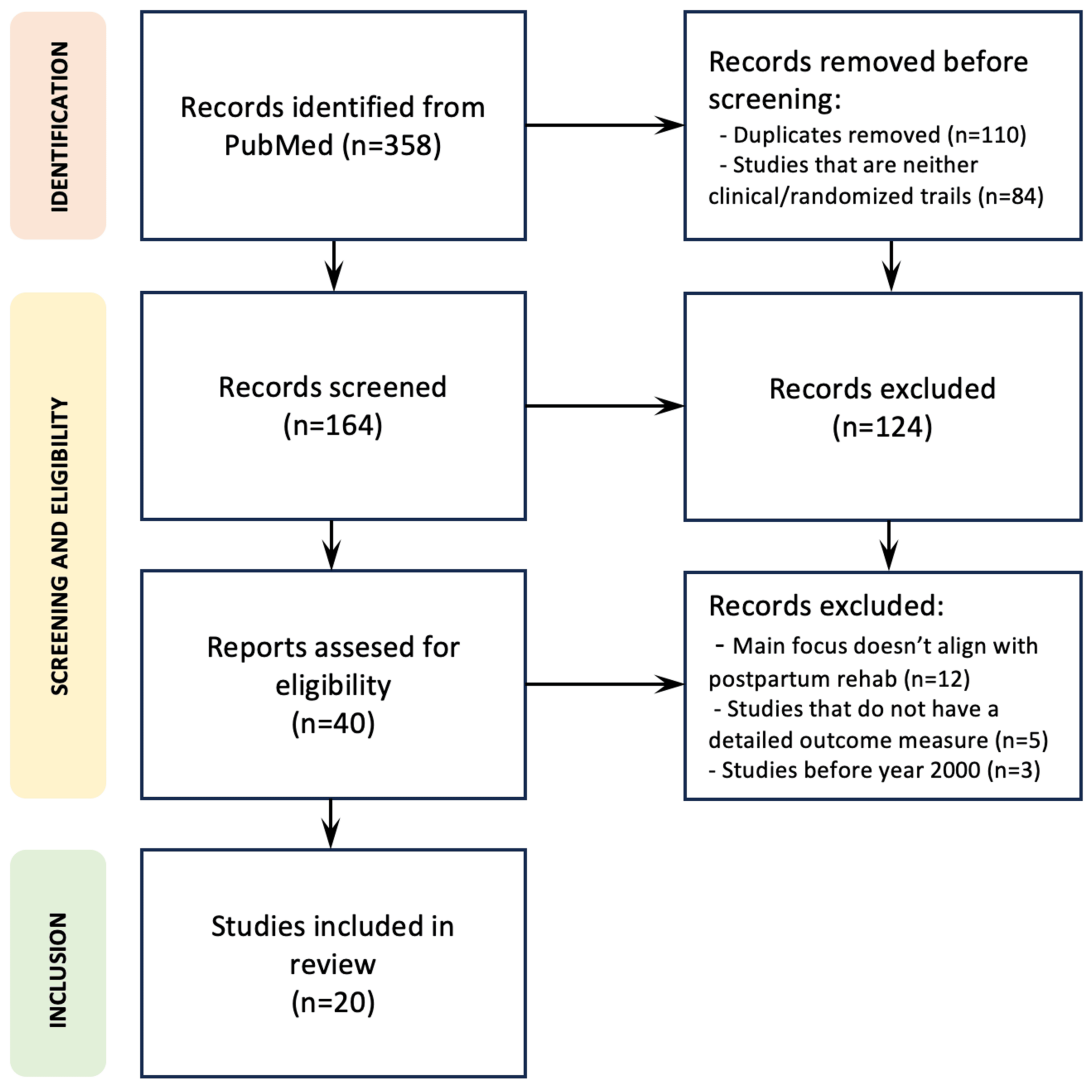
Preferred Reporting Items for Systematic Reviews and Meta-Analyses (PRISMA) diagram showcasing the review methodology.

The literature for this review is systematically gathered from PubMed, employing a comprehensive search strategy using keywords such as "postpartum rehabilitation," "post-delivery rehabilitation," or "post-natal rehabilitation." These search terms are selected to ensure inclusivity and relevance to the scope of the review. The inclusion criteria focused on studies that conduct clinical trials and randomized controlled trials (RCTs) within the domain of postpartum rehabilitation. This criterion is chosen to prioritize evidence-based research methodologies and ensure a robust foundation for the synthesis of findings. The initial search yielded a total of 358 papers from PubMed. Subsequently, a meticulous screening process is employed to assess the relevance of each study. Initially, titles and abstracts are scrutinized to eliminate studies that do not align with the focus on postpartum rehabilitation or do not employ clinical trials or RCTs. Following this, the full texts of the remaining studies are thoroughly reviewed for eligibility. After the screening process, a total of 20 studies met the stringent criteria for inclusion in the review. The review methodology is shown as a Preferred Reporting Items for Systematic Reviews and Meta-Analyses (PRISMA) diagram in Figure 1.

A categorization framework was designed to systematically organize and classify diverse techniques from the literature. The categorization framework includes four categories, namely, exercise-based techniques, technology integration, medical intervention, and multimodal approaches. The exercise-based techniques include studies that are categorized based on the types of exercises utilized in rehabilitation, such as pelvic floor exercises, diastasis recti-specific exercises, high-intensity training, or yoga-based interventions. Studies that incorporate technological advancements such as the latest engineering techniques, mobile applications, and artificial intelligence in rehabilitation programs fall under the technology integration category. Works that make use of drugs and specialized medical treatments come under the medical intervention category. Finally, multimodal works are those that integrate two or more above-mentioned techniques.

Postpartum rehabilitation literature

This section comprehensively presents and categorizes all relevant literature, supplemented with tables containing additional details for works within each category.

Exercise-Based Techniques

The study by Kim et al. [9] aimed to evaluate how effective a real-time video conferencing platform (Zoom) is for exercise intervention in improving various postpartum measures among women with diastasis recti. Thirty-seven participants were randomly assigned to either online (n = 19) or offline (n = 18) groups. The online group participated in 40-minute trunk stabilization exercise sessions twice a week for six weeks via video conferencing, while the offline group attended the same sessions in person. Both groups demonstrated significant improvements in inter-recti distance, abdominal muscle thickness, static trunk endurance, and maternal quality of life. Although the offline group showed a more pronounced improvement, the study suggests that exercise interventions through video conferencing can effectively enhance postpartum outcomes, providing an alternative to in-person interventions.

Hilde et al. [10] aimed to explore the potential of early postpartum pelvic floor muscle training (PFMT) in reducing levator ani (LA) avulsions and decreasing the levator hiatus (LH) area in women who had undergone vaginal delivery. This randomized controlled study involved 175 primiparous women who were randomly assigned to either the PFMT group or the control group. The PFMT group received supervised classes and was instructed to perform daily home-based exercises for 16 weeks. Six months postpartum, both groups showed a decrease in the incidence of complete LA avulsion, although the difference between them was not statistically significant. Furthermore, there were no significant differences observed in the LH area during rest, contraction, or the Valsalva maneuver. The study concluded that early PFMT did not lead to a significant reduction in LA avulsions or the LH area compared to the natural remission process.

The study by ElDeeb et al. [11] aimed to evaluate the effects of stabilizing exercises with and without pelvic floor muscle (PFM) training on pelvic girdle pain (PGP), functional disability, trunk range of motion (ROM), and PFM strength in postpartum women. Forty participants were randomly divided into two groups: Group A, which received local stabilizing exercises, and Group B, which received stabilizing exercises combined with PFM training. Both groups demonstrated a significant reduction in pain and functional disability, as well as a notable increase in trunk ROM and PFM strength. However, Group B exhibited a more pronounced decrease in pain and functional disability, along with a greater improvement in PFM strength compared to Group A. The study suggests that incorporating PFM training into rehabilitation programs for postpartum PGP is crucial for optimal outcomes.

The study conducted by Kim et al. [12] sought to examine the effectiveness of PFM exercises with trunk stabilization in treating postpartum urinary incontinence, comparing supervised and unsupervised training methods. Eighteen participants were randomly allocated to either a supervised or an unsupervised training group. The supervised group, receiving verbal guidance from a physiotherapist, demonstrated more substantial improvements in urinary symptoms, quality of life, total score, maximal vaginal squeeze pressure, and holding time compared to the unsupervised group. These findings indicate that supervised PFM exercises with trunk stabilization may offer greater benefits in managing postpartum urinary incontinence. A summary of exercise-based techniques is shown in Table 1.

**Table 1:** Overview of the works that use exercise-based techniques. RCT: randomized controlled trial

Reference	Groups	Population size	Study type	Study duration	Delivery type	Parity	Year
Kim et al. [9]	Intervention group: received 40-minute trunk stabilization twice a week via video conferencing. Control group: received the same training in person	37	RCT	6 weeks	Any	Any	2022
Hilde et al. [10]	Intervention group: received supervised pelvic floor training once a week and home-based training daily. Control group: received no intervention	175	RCT	16 weeks	Vaginal	Primiparous	2023
ElDeeb et al. [11]	Group A: received local stabilizing exercises. Group B: received local stabilizing exercises along with pelvic floor training	40	RCT	12 weeks	Any	Any	2019
Kim et al. [12]	Supervised group: received verbal instruction from the physiotherapist to perform pelvic floor muscle exercises. Unsupervised group: received no intervention	18	RCT	—	Any	Any	2012

Technology Integration

The study described by Li et al. [13] evaluates the effects of different electrical stimulation protocols on postpartum women with severely weakened PFM strength. Sixty-seven participants were randomly divided into two groups: Group A received transvaginal electrical stimulation (TVES) for five sessions, while Group B underwent TVES for three sessions combined with electromyogram (EMG)-triggered neuromuscular stimulation twice. Results indicated improvements in pelvic muscle strength for both groups, with Group A demonstrating significant enhancements in EMG variables related to contractile amplitude and stability. Additionally, the incidence of correct PFM contraction increased in Group A after five treatments. The study suggests that for postpartum women with severely weakened muscle strength, five sessions of TVES may be more effective in improving pelvic muscle control and strength, even within a short treatment period.

The study detailed by Liang et al. [14] aimed to evaluate the effects of a comprehensive rehabilitation program on rectus diastasis (RD) closure and quality of life in postpartum women. In an RCT involving 66 participants, the study group underwent electromyographic-biofeedback-assisted pelvic floor muscle training (BAPFMT) combined with neuromuscular electrical stimulation (NMES) of the rectus abdominis, while the control group received NMES alone. The results revealed a significant reduction in inter-recti distance (IRD) in the study group, indicating RD closure. Moreover, the study group demonstrated a notable improvement in the physical domain of quality of life compared to the control group. These findings suggest that implementing a postpartum program incorporating BAPFMT is both feasible and beneficial for women with RD.

The study conducted by Yang et al. [15] aimed to investigate the postoperative effects of auricular acupuncture on primiparas who underwent cesarean sections, with the goal of enhancing postpartum rehabilitation. A total of 120 primiparas were randomly allocated into either an observation group or a control group. Both groups received routine post-surgery care, while the observation group additionally underwent auricular acupuncture. Results indicated that the observation group exhibited significantly lower rates of postpartum pain, earlier restoration of bowel function, reduced incidence of complications, and shorter hospital stays compared to the control group. The study concluded that auricular acupuncture effectively alleviates postpartum pain, facilitates recovery, and diminishes the likelihood of complications, thereby contributing to postpartum rehabilitation.

The study described by Yin et al. [16] aimed to evaluate the utility of ultrasound technology and rehabilitation training based on an artificial intelligence algorithm in postpartum recovery from pelvic organ prolapse. Sixty patients with mild to moderate pelvic organ prolapse were randomly assigned to either an experimental group (receiving rehabilitation training) or a control group (receiving routine guidance without training). After one and three months of treatment, the experimental group exhibited significantly greater levator ani muscle thickness, reduced anterior and posterior diameter of the perineal hiatus, and increased PFM strength compared to the control group. Ultrasound images processed using the artificial intelligence algorithm demonstrated enhanced clarity and quality, with superior performance metrics compared to traditional methods. The study concluded that the integration of artificial intelligence algorithm-based ultrasound technology with pelvic floor rehabilitation training is effective for postpartum care in patients with pelvic organ prolapse.

The study detailed by Chen et al. [17] aimed to investigate the potential utility of an image enhancement algorithm in assessing pelvic floor rehabilitation training for preventing postpartum female pelvic floor dysfunction (FPFD). Seventy patients with FPFD were randomly allocated into two groups: a control group receiving standard nursing care and an experimental group receiving pelvic floor rehabilitation training in addition to standard care. Ultrasound images were subjected to processing using an image enhancement algorithm, and the effectiveness was evaluated using the International Consultation on Incontinence Questionnaire-Short Form (ICIQ-SF) and Pelvic Floor Distress Inventory-20 (PFDI-20). The results indicated a significant improvement in image quality following processing with the image enhancement algorithm. The diagnostic accuracy for FPFD increased from 73.34% to 89.86%. Moreover, the clinical response rate in the experimental group surpassed that of the control group. Both groups demonstrated reduced scores on the ICIQ-SF and PFDI-20 following rehabilitation training. The study suggests promising applications for the image enhancement algorithm in evaluating pelvic floor rehabilitation training aimed at preventing postpartum FPFD.

The study presented by Uzunkaya et al. [18] sought to investigate the impact of kinesio taping (KT) on acute pain, breastfeeding behavior, and comfort levels in women undergoing cesarean section. Conducted as a randomized, single-blinded trial, 48 participants were assigned to either the KT group (receiving KT application on breasts and rectus abdominis muscles) or the control group. Assessments utilizing the Visual Analogue Scale (VAS), LATCH, Postpartum Breastfeeding Self-Efficacy Scale (PBSES), and Postpartum Comfort Scale (PCS) were performed on postoperative days 0, 1, and 2. Findings indicated that the KT group experienced significantly reduced pain, enhanced breastfeeding self-efficacy, and improved postpartum comfort compared to the control group. The study suggests that KT yields positive effects on pain management, breastfeeding success, and comfort levels in women undergoing cesarean section.

The RCT trial documented by Yang et al. [19] aimed to evaluate the effects of rehabilitation exercises in conjunction with Direct Vagina Low Voltage Low-Frequency Electric Stimulation (DES) on pelvic nerve electrophysiology and tissue function following childbirth. The study involved 189 primiparous women who had undergone episiotomy or second-degree episiotomy tear, categorized into three groups: control, training, and combination. After a three-month period, variations were noted among the groups in terms of pelvic organ prolapse, incontinence score, PFM strength, and PFM electrophysiology. The findings suggested that rehabilitation exercises, particularly when combined with DES, had favorable outcomes in promoting postpartum pelvic nerve tissue recovery and enhancing the overall quality of life for women.

The study conducted by Wang et al. [20] aimed to evaluate the clinical efficacy of static and dynamic magnetic resonance imaging (MRI) in assessing the effectiveness of electrical stimulation combined with biofeedback therapy for postpartum pelvic organ prolapse. Fifty primiparas were divided into a treatment group, which received the therapy, and a control group, which experienced spontaneous recovery. Following six weeks of treatment, the treatment group exhibited a significantly higher rate of effectiveness in addressing pelvic organ prolapse. Dynamic MRI scans revealed improvements in the thickness of bilateral puborectal muscles and various anatomical parameters in the treatment group compared to the control group at 12 weeks postpartum. These findings suggest that electrical stimulation combined with biofeedback therapy can enhance recovery from pelvic organ prolapse, and static and dynamic MRI serve as valuable tools for objectively assessing the effects of pelvic floor rehabilitation.

The protocol outlined by Oblasser et al. [21] aims to conduct a feasibility trial as preparation for a future RCT, which will assess the effectiveness of vibrating vaginal pelvic floor training balls for postpartum PFM rehabilitation. The trial will involve 56 postpartum women in Vienna, who will be randomly allocated to either the vibrating vaginal balls group or the standard PFM exercise group for a duration of 12 weeks. The study will assess recruitment strategies, randomization procedures, intervention adherence, and data collection methods. Outcome measures will include evaluations of PFM performance reported by participants and perineometry assessments. The results of this feasibility trial will inform the design of the subsequent full RCT and provide valuable insights into women’s experiences with the interventions and participation in the study. A summary of works that fall under the technology integration category is shown in Table 2.

**Table 2:** Overview of the works that incorporate technological advancements. RCT: randomized controlled trial

Reference	Groups	Population size	Study type	Study duration	Delivery type	Parity	Year
Li et al. [13]	Group A: received transvaginal electrical stimulation five times. Group B: received transvaginal electrical stimulation three times and electromyogram triggered neuromuscular stimulation two times	67	RCT	—	Any	Any	2020
								Liang et al. [14]	Intervention group: received electromyographic biofeedback-assisted pelvic floor muscle training combined with neuromuscular electrical stimulation of the rectus abdominis. Control group: only received neuromuscular electrical stimulation	66	RCT	6 weeks	Any	Any	2022
Yang et al. [15]	Intervention group: received routine post-surgery care and auricular acupuncture. Control group: only received routine post-surgery care	120	RCT	5 days	C-section	Primiparous	2019								
								Yin et al. [16]	Intervention group: received pelvic floor rehabilitation training. Control group: received routine guidance without training	60	RCT	1 - 3 months	Any	Any	2022
Chen et al. [17]	Intervention group: received routine nursing and pelvic floor rehabilitation training. Control group: only received routine nursing	70	RCT	—	Any	Any	2022
								Uzunkaya et al. [18]	Intervention group: kinesio taping was applied to breasts and rectus abdominis muscles. Control group: no taping	48	RCT	3 days	C-section	Any	2023
Yang et al. [19]	Combination group: received vagina low voltage electrical stimulation plus rehabilitation exercises. Training group: received rehabilitation exercises. Control group: received routine postpartum guidance	189	RCT	3 months	Any	Primiparous	2017
								Wang et al. [20]	Intervention group: received electrical stimulation and biofeedback therapy. Control group: received no intervention	50	RCT	6 - 12 weeks	Any	Primiparous	2019
																Oblasser et al. [21]	Intervention group: used vibrating vaginal balls. Control group: received pelvic floor muscle training	56	RCT	12 weeks	Any	Any	2016

Multimodal Approaches

The study described by Wang et al. [22] aimed to evaluate the effects of a rehabilitation program on lumbopelvic pain following childbirth. In an RCT involving postpartum women experiencing lumbopelvic pain, the intervention group (n = 48) underwent PFM training combined with neuromuscular electrical stimulation of paraspinal muscles for 12 weeks, while the control group (n = 48) received only neuromuscular electrical stimulation for the same duration. The results indicated significant improvements in pain, disability, and the physical domain of quality of life among participants in the intervention group at both six and 12 weeks compared to the control group. The study concluded that implementing a postpartum program for women with lumbopelvic pain is feasible and contributes to enhancing the physical aspect of quality of life.

The study conducted by Bhat et al. [23] aimed to compare the effectiveness of combining sexually induced orgasms with Kegel exercises versus Kegel exercises alone as a treatment method to enhance postpartum PFM strength and sexual function in primiparous women who underwent uncomplicated vaginal deliveries. This randomized two-arm study involved sexually active primiparous women, with Group 1 performing daily Kegel exercises and Group 2 engaging in self-initiated/partnered sexual activity-induced orgasms along with daily Kegel exercises. The results revealed that sexual function and the ability to relax the pelvic floor were significantly better in Group 2 compared to Group 1 at each monthly assessment interval. In addition, PFM strength during voluntary contraction was significantly higher in Group 2 at the end of the six-month period. The study suggests that incorporating sexually induced orgasms into postpartum pelvic floor rehabilitation can significantly enhance muscle strength and sexual function in primiparous women who have undergone uncomplicated vaginal deliveries.

The study detailed by Xue et al. [24] explores the implementation of a multidisciplinary team Enhanced Recovery After Surgery (MDT-ERAS) model in cesarean section procedures and evaluates its health-economic advantages. A cohort of 572 pregnant women undergoing cesarean section was randomly allocated into either an experimental group (managed with MDT-ERAS) or a control group (treated with conventional rehabilitation methods). The MDT-ERAS group exhibited reduced pain scores, shorter recovery periods, decreased incidences of postpartum hemorrhage and neonatal complications, and lower hospitalization expenses. These findings suggest that MDTERAS enhances postoperative recovery and maternal and neonatal outcomes and offers superior health and economic advantages compared to traditional rehabilitation approaches.

The objective of Adnan et al. [25] was to examine the effects of a static exercise regimen compared to Swiss ball training on the core muscles of the lower back and pelvic region in postpartum patients experiencing low back pain. In this RCT, 30 patients participated, with 15 assigned to each group. Both exercise protocols, involving static exercises and Swiss ball training, demonstrated notable enhancements within their respective groups. However, there was no significant discrepancy observed between the two groups. Consequently, the study concluded that both exercise programs were equally efficacious in the rehabilitation of postpartum low back pain.

The pilot study outlined by Gausel et al. [26] aimed to assess the feasibility of a comparative investigation between the impact of individualized rehabilitation combined with chiropractic treatment and individualized rehabilitation alone in women experiencing persistent one-sided pelvic girdle pain (PGP) between three and six months post-delivery. Initially, 330 women who had reported pelvic pain during pregnancy were recruited, among whom 13 were diagnosed with predominant one-sided PGP and subsequently enrolled in the study. Six participants were randomly assigned to receive individualized rehabilitation along with chiropractic treatment, while five received individualized rehabilitation alone. Both groups demonstrated improvements in disability and pain over the course of 20 weeks, with no occurrence of serious adverse events. The study suggests that exploring the effectiveness of chiropractic treatment for managing PGP pain is feasible, albeit with careful consideration of patient recruitment conditions.

The multicenter prospective RCT detailed by Sun et al. [27] aimed to assess the influence of postpartum pelvic floor rehabilitation on pelvic floor electrical physiological indexes and the prevention of female pelvic floor dysfunction in China. The study involved 324 postpartum women who were randomly allocated to either treatment or control groups. The treatment group received electrical stimulation and biofeedback treatment, while the control group performed PFM exercises at home. The results obtained at six and 12 months postpartum revealed noteworthy enhancements in pelvic floor electrical physiological indexes among participants in the treatment group compared to those in the control group. The therapy was observed to augment PFM fiber strength and mitigate pelvic floor dysfunction, with no significant impact observed on quality of life or sexual functioning. A summary of multimodal works is shown in Table 3.

**Table 3:** Overview of the works that use the multimodal approach. RCT: randomized controlled trial

Reference	Groups	Population size	Study type	Study duration	Delivery type	Parity	Year
Wang et al. [22]	Intervention group: received pelvic floor muscle training along with neuromuscular electrical stimulation. Control Group: only received neuromuscular electrical stimulation	96	RCT	6-12 weeks	Any	Any	2021
Bhat et al. [23]	Intervention group: performed kegel exercises and were involved in self/partner-initiated sexual orgasm. Control group: only performed kegel exercises	55	RCT	6 months	Vaginal	Primiparous	2022
Xue et al. [24]	Intervention group: was treated with a multi-disciplinary team enhanced recovery after surgery technique. Control group: was treated with traditional rehabilitation methods	572	RCT	—	C-section	Any	2019
								Adnan et al. [25]	Group A: received static core exercises Group B: received Swiss ball training	30	RCT	—	Any	Any	2021
Gausel et al. [26]	Intervention group: received individualized rehabilitation and chiropractic treatment. Control group: only received individualized rehabilitation	13	RCT	20 weeks	Any	Any	2019								
								Sun et al. [27]	Intervention group: received electrical stimulation and bio-feedback treatment. Control group: received pelvic floor muscle exercise at home	324	RCT	6-12 months	Any	Any	2015

Medical Interventions

The prospective, double-blind, RCT outlined by Lalmand et al. [28] examined the effects of intrathecal analgesia and continuous wound infiltration with ropivacaine following cesarean delivery. The primary outcome measure was the duration of analgesia, defined as the time until the first request for morphine. Secondary outcomes included the cumulative consumption of morphine postoperatively, adverse effects, and the time until the first ambulation. The study enrolled 192 parturients, who were allocated into control, morphine, and catheter groups. Both intrathecal morphine administration and continuous wound infusion of ropivacaine significantly prolonged postoperative analgesia duration and efficacy compared to the control group. In addition, cumulative morphine consumption was lower in the morphine and catheter groups, without an increase in adverse effects.

Discussion

The systematic review presented in this paper delves into the recent trends in postpartum rehabilitation, categorizing the existing literature into four distinct domains: exercise-based techniques, technology integration, medical interventions, and multi-modal approaches. Through this comprehensive analysis, a nuanced understanding of the current landscape of postpartum care emerges, highlighting key interventions and their respective impacts on recovery and functional outcomes.

In the realm of exercise-based techniques, pelvic floor exercises [10,11], trunk stabilization exercises [9], and physiotherapy [12] emerge as cornerstone modalities for postpartum rehabilitation. The consistent utilization of these techniques across various studies underscores their recognized efficacy in addressing common postpartum challenges, such as pelvic floor dysfunction and core instability. The range of population sizes (20-200 participants) and study durations (six weeks to 12 weeks) reflects the robust evidence base supporting the effectiveness of exercise-based interventions in promoting recovery and improving functional outcomes among postpartum individuals.

Technology integration emerges as a dominant theme in postpartum rehabilitation, with a diverse array of innovative techniques showcased in the literature. Electrical stimulation [13], biofeedback [14], acupuncture [15], and vibrating vaginal balls [21] are among the most commonly utilized technologies, offering novel approaches for enhancing rehabilitation outcomes and improving patient engagement. The variability in population sizes (ranging from 50 to 200 participants) and study durations (ranging from three days to three months) underscores the versatility and adaptability of technological interventions in addressing the evolving needs of postpartum individuals.

Multimodal approaches emerge as a particularly promising avenue for optimizing postpartum rehabilitation outcomes, capitalizing on the synergistic effects of combining various techniques and modalities. By integrating technology, artificial intelligence, image processing, and exercise-based interventions, multimodal approaches offer a comprehensive and personalized approach to postpartum care that addresses the diverse needs and preferences of individuals. The wide range of population sizes (ranging from 10 to 500 participants) and study durations (ranging from six weeks to 12 months) reflects the versatility and adaptability of multi-modal interventions in tailoring treatment plans to individual needs and achieving sustained improvements in functional outcomes. While the majority of the rehabilitation works do not distinguish the type of delivery, specialized techniques for vaginal and cesarean-section deliveries have also been explored. Works, such as [10,23], show that specialized pelvic floor and kegel exercises are beneficial. In the case of cesarean-section deliveries, techniques such as auricular acupuncture [15], kinesio taping [18], and MDT-ERAS [24] are helpful.

Medical interventions constitute a smaller subset of the reviewed literature but present promising avenues for enhancing postpartum recovery and alleviating discomfort following childbirth. The identification of intrathecal analgesia and continuous ropivacaine administration [28] as notable medical interventions highlights the potential for pharmacological approaches to complement existing rehabilitation modalities and improve patient outcomes. Despite the limited number of studies in this category, the inclusion of medical interventions underscores the importance of a multidisciplinary approach to postpartum care that considers the integration of pharmacological and rehabilitation strategies.

In summary, this systematic review provides a comprehensive overview of recent trends in postpartum rehabilitation, highlighting the diversity of approaches and interventions aimed at promoting recovery and improving quality of life among postpartum individuals. Moving forward, continued research and innovation in this field are essential to further elucidate the efficacy of various interventions and develop comprehensive, evidence-based strategies for optimizing postpartum care. A full tabular summary of all studies included in this review is highlighted in Table 4.

**Table 4:** A summary of all the works that are reviewed in this study. RCT: randomized controlled trial

Reference	Groups	Population size	Study type	Study duration	Delivery type	Parity	Year
Kim et al. [9]	Intervention group: received 40-minute trunk stabilization twice a week via video conferencing. Control group: received the same training in person	37	RCT	6 weeks	Any	Any	2022
Hilde et al. [10]	Intervention group: received supervised pelvic floor training once a week and home-based training daily. Control group: received no intervention	175	RCT	16 weeks	Vaginal	Primiparous	2023
ElDeeb et al. [11]	Group A: received local stabilizing exercises. Group B: received local stabilizing exercises along with pelvic floor training	40	RCT	12 weeks	Any	Any	2019
Kim et al. [12]	Supervised group: received verbal instruction from the physiotherapist to perform pelvic floor muscle exercises. Unsupervised group: received no intervention	18	RCT	—	Any	Any	2012
								Li et al. [13]	Group A: received transvaginal electrical stimulation five times. Group B: received transvaginal electrical stimulation three times and electromyogram triggered neuromuscular stimulation two times	67	RCT	—	Any	Any	2020
Liang et al. [14]	Intervention group: received electromyographic biofeedback-assisted pelvic floor muscle training combined with neuromuscular electrical stimulation of the rectus abdominis. Control group: only received neuromuscular electrical stimulation	66	RCT	6 weeks	Any	Any	2022								
								Yang et al. [15]	Intervention group: received routine post-surgery care and auricular acupuncture. Control group: only received routine post-surgery care	120	RCT	5 days	C-section	Primiparous	2019
Yin et al. [16]	Intervention group: received pelvic floor rehabilitation training. Control group: received routine guidance without training	60	RCT	1-3 months	Any	Any	2022
								Chen et al. [17]	Intervention group: received routine nursing and pelvic floor rehabilitation training. Control group: only received routine nursing	70	RCT	—	Any	Any	2022
Uzunkaya et al. [18]	Intervention group: kinesio taping was applied to breasts and rectus abdominis muscles. Control group: no taping	48	RCT	3 days	C-section	Any	2023								
Yang et al. [19]	Combination group: received vagina low voltage electrical stimulation plus rehabilitation exercises. Training group: received rehabilitation exercises. Control group: received routine postpartum guidance	189	RCT	3 months	Any	Primiparous	2017
Wang et al. [20]	Intervention group: received electrical stimulation and biofeedback therapy. Control group: received no intervention	50	RCT	6-12 weeks	Any	Primiparous	2019
								Oblasser et al. [21]	Intervention group: used vibrating vaginal balls. Control group: received pelvic floor muscle training	56	RCT	12 weeks	Any	Any	2016
Wang et al. [22]	Intervention group: received pelvic floor muscle training along with neuromuscular electrical stimulation. Control Group: only received neuromuscular electrical stimulation	96	RCT	6-12 weeks	Any	Any	2021								
Bhat et al. [23]	Intervention group: performed kegel exercises and were involved in self/partner-initiated sexual orgasm. Control group: only performed kegel exercises	55	RCT	6 months	Vaginal	Primiparous	2022
Xue et al. [24]	Intervention group: was treated with a multi-disciplinary team enhanced recovery after surgery technique. Control group: was treated with traditional rehabilitation methods	572	RCT	—	C-section	Any	2019
								Adnan et al. [25]	Group A: received static core exercises. Group B: received Swiss ball training	30	RCT	—	Any	Any	2021
Gausel et al. [26]	Intervention group: received individualized rehabilitation and chiropractic treatment. Control group: only received individualized rehabilitation	13	RCT	20 weeks	Any	Any	2019								
								Sun et al. [27]	Intervention group: received electrical stimulation and biofeedback treatment. Control group: received pelvic floor muscle exercise at home	324	RCT	6-12 months	Any	Any	2015
Lalmand et al. [28]	Catheter group: received NaCl intrathecally and ropivacaine infused in a catheter. Morphine group: received morphine intrathecally and NaCl through a catheter. Control group: received NaCl intrathecally and through a catheter	192	CT	—	Any	Any	2017								

Risk-of-Bias Assessment

**Figure 2:**
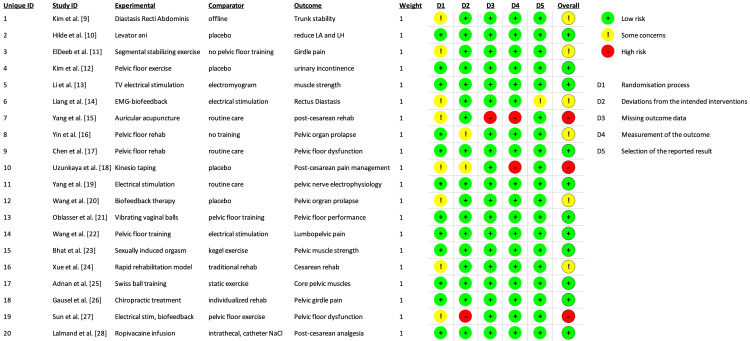
Risk-of-bias assessment of all studies included in this review.

**Figure 3:**
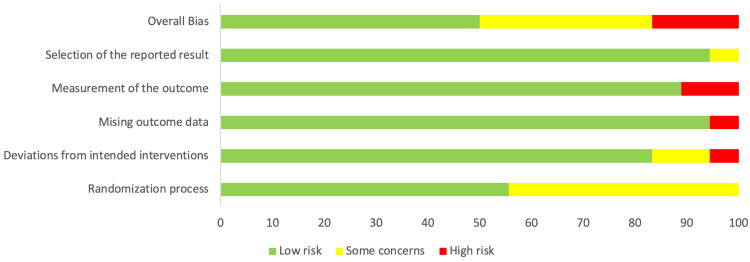
Overview of the risk-of-bias analysis results.

Risk-of-bias (RoB) assessment was conducted on all the studies included in this review using the RoB 2 tool [29]. The RoB results are shown as a traffic light plot in Figure 2. The analysis revealed a very low overall bias, as shown in Figure 3.

## Conclusions

This systematic review examined postpartum rehabilitation through approximately 20 studies out of 358, categorized into exercise-based interventions, technology integration, medical interventions, and multimodal approaches. Pelvic floor training emerged as the most common method, with trunk stabilization exercises and electrical stimulation also highlighted for restoring pelvic and core strength. In addition, innovative techniques like chiropractic treatments, Kinesio taping, and acupuncture showcased the evolving landscape of postpartum care. This review provides valuable insights into effective postpartum rehabilitation strategies, guiding clinical practice, and future research to optimize postpartum recovery and maternal well-being. The findings emphasize the importance of personalized rehabilitation programs. Continued exploration of diverse approaches will be crucial in addressing the varied needs of postpartum women.
